# *De novo* RNA sequencing transcriptome of *Rhododendron obtusum* identified the early heat response genes involved in the transcriptional regulation of photosynthesis

**DOI:** 10.1371/journal.pone.0186376

**Published:** 2017-10-23

**Authors:** Linchuan Fang, Jun Tong, Yanfang Dong, Dongyun Xu, Jing Mao, Yuan Zhou

**Affiliations:** Institute of Fruit & Forest Tree, Wuhan Academy of Agricultural Science & Technology, Wuhan, Hubei, China; Key Laboratory of Horticultural Plant Biology (MOE), CHINA

## Abstract

*Rhododendron spp*. is an important ornamental species that is widely cultivated for landscape worldwide. Heat stress is a major obstacle for its cultivation in south China. Previous studies on rhododendron principally focused on its physiological and biochemical processes, which are involved in a series of stress tolerance. However, molecular or genetic properties of rhododendron’s response to heat stress are still poorly understood. The phenotype and chlorophyll fluorescence kinetics parameters of four rhododendron cultivars were compared under normal or heat stress conditions, and a cultivar with highest heat tolerance, “Yanzhimi” (*R*. *obtusum*) was selected for transcriptome sequencing. A total of 325,429,240 high quality reads were obtained and assembled into 395,561 transcripts and 92,463 unigenes. Functional annotation showed that 38,724 unigenes had sequence similarity to known genes in at least one of the proteins or nucleotide databases used in this study. These 38,724 unigenes were categorized into 51 functional groups based on Gene Ontology classification and were blasted to 24 known cluster of orthologous groups. A total of 973 identified unigenes belonged to 57 transcription factor families, including the stress-related HSF, DREB, ZNF, and NAC genes. Photosynthesis was significantly enriched in the Kyoto Encyclopedia of Genes and Genomes pathway, and the changed expression pattern was illustrated. The key pathways and signaling components that contribute to heat tolerance in rhododendron were revealed. These results provide a potentially valuable resource that can be used for heat-tolerance breeding.

## Introduction

Rhododendron (*Rhododendron spp*. L.) is one of the most popular woody flowers worldwide; more than 800 rhododendron species are diploid with 2n = 2x = 26 and with a monoploid genome size ranging from 0.67 pg to 0.83 pg [1]. In temperate and tropical zones, a greater risk of heat stress occurs in the introduction and cultivation of rhododendron plants. Four rhododendron species showed the highest absolute and relative net photosynthetic rates in response to increasing temperatures coupled with the highest photosynthetic rates at 25°C [2]. An evaluation of growth and survival of rhododendron in warm climates suggests the considerable differences in heat tolerance among species. Few species of rhododendron can survive under warm conditions. For example, *R*. *hyperythrum* is native to Taiwan at elevations of 900–1200 m [3]; this plant grows well in the warm climate of Louisiana [4] and shows good performance in greenhouse evaluations of heat tolerance among rhododendron species [5]. By contrast, *R*. *russutum* is an alpine, lepidote rhododendron that is part of the Laponicum series; it is native to the northwest Yunnan province of China at elevations of 3400–4300 m and grows poorly in warm climate [6].

Global challenges, such as climate change, environmental degradation, and toxic waste, subject plants to various stresses during growth. Drought, high salinity, and extreme temperature are the major limiting factors of higher plant growth and production. General heat stress causes multifarious and often adverse alterations in plant growth, development, physiological processes, and yield [7]. Overheating can lead to lethal heat limits, thereby resulting in heat-related injury or death [8]. Plants continuously struggle for survival under various environmental stress conditions, including heat stress. A plant tolerates heat stress partially through physical changes within the plant body and by initiating signals to change the metabolism. Particularly, the plants produce compatible solutes that can organize the structures of proteins and cellular components. The leaf photosynthetic system is particularly sensitive to high-temperature stress sites [9], and photosynthesis system II (PSII) is the activity center of the vital processes of photosynthesis in the most sensitive aspects, particularly at high temperature. Therefore, chlorophyll fluorescence (ChlF) parameters are increasingly used as effective tools for the analysis of plant PSII function under high temperature stress [10, 11].

Numerous genes in higher plants are activated in response to the abovementioned abiotic stresses. Genes can either directly respond to stresses or regulate the expression of other genes and signal transcription. Transcription factors (TFs) function in gene expression by combining the DNA-binding and cis-acting elements. Many TFs, such as AP2/DREB (dehydration-responsive element-binding protein), NAC (NAM in petunia, CUP-SHAPED COTYLEDON2 (CUC2) and NAP), HSF (Heat Shock Transcription Factor), bZIP (basic leucine zipper protein), and WRKY, are related to stress resistance in plants. For example, HsfA1s act as the “master regulators” in heat stress regulatory network and are necessary for the activation of transcriptional networks, including DREB2A, HsfA2, HsfA7a, HsfBs, and MBF1C [12]. DREB2A is a candidate protein that binds to the DRE sequence and regulates the expression of drought stress-responsive genes [13, 14]. NAC019 is a novel TF that regulates heat stress response, binds to the promoters of heat stress responsive TFs, and positively affects the expression of some heat stress-inducible genes by interacting with its partner RCF2 [15]. These TFs interact to regulate the downstream gene expressions, including heat shock proteins (HSPs) and reactive oxygen species (ROS). ROS scavenging enzymes are major functional proteins that are induced by HS and are well-known target genes of HS-responsive TFs [16]. HSPs include HSP100, HSP90, HSP70, HSP60, and small HSPs (sHSPs). HSPs regulate the protein quality by renaturing a variety of proteins that were denatured by HS. Chloroplast HSP21 interacts with plastid nucleoid protein pTAC5 and is essential for chloroplast development under heat stress [17].

Only 3 gene locus and 2285 expressed sequence tags (ESTs) can be found for rhododendrons in the GenBank (National Center for Biotechnology Information). Minimal information was found on the functional genes in *Rhododendron* spp. The improved transformation efficiency of some rhododendron plants can accelerate the generation of genetically modified (GM) rhododendron cultivars [18, 19]. Some physiological studies revealed that SA and CaCl_2_ might interact to alleviate the heat stress in rhododendrons [20]. However, the lack of genomic sequence information, especially for the functional genes involved in the formation of importance traits, remains a major obstacle that severely restricts the future application of molecular-assisted selection (MAS) or GM breeding.

Considering that many species of rhododendron are important ornamental plants, a better understanding of the heat tolerance of different species and provenances and the specific characteristics and molecular mechanisms that influence heat tolerance are necessary for the MAS and the improvement of rhododendron breeding in different adverse environments. The objectives of this study were as follows: 1) to assess the heat tolerance ability of the commonly used rhododendron varieties in South China and select a suitable material for the executive RNA sequencing; and 2) to achieve the expression profiles and unigene sequences, especially for the TF regulator involved in the stress response, by using the RNA sequencing transcriptome, which assists in further gene cloning and for conducting functional research on molecular mechanisms underlying different heat response variations in different species. Among the 92,463 unigenes in transcriptome, 38,724 unigenes with annotation were identified. The results from this study provide abundant genomic information for future research and clarify the molecular mechanisms governing the heat responsive process involved in the transcriptional regulation of photosynthesis.

## Methods

### Plant materials and growth conditions

Samples of rhododendron “Yanzhimi” (*R*. *obtusum*), “Xiajinpao” (*R*. *hybridum*), “Jinpao” (*R*. *hybridum*), and “Yanchun” (*R*. *hybridum*) were used as experimental materials. Two-year-old self-rooting plants were transferred and grown in the greenhouse for 3 months in Wuhan Academy of Agricultural Sciences and Technology. Control plants were then grown in a growth chamber under continuous light (20–40 μm sec^-1^ m^-2^) at 25°C. For heat treatments, the plants were transferred to 40°C under the same light source. Fresh leaves were collected after 4 and 24 h of treatment. Tissue from 15 randomly selected mature leaves were pooled to minimize biological variability for each sample and frozen in liquid nitrogen. Samples of leaves were separated and frozen in liquid nitrogen for further analysis, and three biological replicates were set for each time point.

### Measurement of chlorophyll fluorescence parameters

The third fully expanded leaf attached to the plant was subjected to a pulse−amplitude modulation fluorometer (PAM–2500, Walz, Germany) to detect the chlorophyll fluorescence parameters. After 20 min of dark adaptation, the minimum fluorescence level (*F*o) was determined under low-intensity measuring light. Maximum fluorescence level (*F*m) was measured after 0.5 s saturating pulse at 4,000 μmol m^−2^ s^−1^. Steady-state fluorescence level (*F*s) was obtained after 20 min of actinic light (234 μmol m^−2^ s^−1^) adaptation. Light-adapted maximum fluorescence level (*F*m’) was measured with a second saturating pulse (0.5 s, 4,000μmol m^−2^ s^−1^). The actinic light was then closed, and the light-adapted minimum fluorescence level (*F*o’) was determined using a far-red light for 5 s. These parameters were used to obtain four identification indexes, as follows: *F*v/*F*m = (*F*m−*F*o)/Fm, *F*v’/*F*m’ = (*F*m’-*F*o’)/*F*m’ [21]. The parameters *F*v/*F*m and *F*v’/*F*m’ represent the maximum photochemical quantum yield of PS II and the excitation energy capture efficiency of PSII reaction center, respectively. The survival rate of 30 leaves was observed and statistically calculated for each sample with three biological and technical repeats.

### RNA isolation and construction of mRNA-Seq library

Total RNA was isolated using TRIzol reagent (Invitrogen, Carlsbad, CA) according to the manufacturer's instructions. The RNA quality was evaluated using Bioanalyzer 2100 (Agilent Technologies, CA, USA), and the A260/A280 ratios of all samples varied from 2.0 to 2.1. Poly(A) + mRNA was purified with oligo(dT) beads. The mRNA was randomly segmented into small fragments by the divalent cations (Fragmentation Buffer, Illumina, Hayward, CA) at 94°C for 5 min, which were then used as templates to synthesize the first-strand cDNA using random hexamer primers. The second-strand cDNA was synthesized using RNAseH and DNA polymerase I. Short cDNA fragments were purified with QiaQuick PCR extraction kit. The cDNA fragments were connected with sequencing adapters according to Illumina’s protocol (San Diego, CA USA). After agarose gel electrophoresis, the target fragments of 300–500 bp were selected for PCR amplification to create the final cDNA library. All of these raw data have been deposited and released in the National Center for Biotechnology Information (NCBI) Sequence Read Archive (http://www.ncbi.nlm.nih.gov/sra/) under bioproject accession PRJNA398170.

### Processing and mapping of Illumina reads

Nine cDNA samples from the control and from 4 and 24 h heat-treated plants were prepared for sequencing. The library was sequenced using Illumina HiSeq X Ten. Raw reads were cleaned by removing the adaptor sequences. Empty reads and reads with unknown sequences “N” were removed before data analysis. All the following analysis were built on getting a precise and unique trascript sequence. So in order to obtain the sequence, we separately subjected cleaned reads to *de novo* assembly using Trinity software (http://trinityrnaseq.sourceforge.net/) with default k mer size set to 25 bp [22]. The Trinity software carried out the assembly by three steps. Firstly, the clean reads were first assembled to produce longer fragments named as trinity. Secondly, the paired-end reads were used to calculate the distance and relation among these transcripts and to avoid obtaining trinities from the same transcript. Finally, the transcripts were connected until they cannot be extended on either end. These sequences were subjected to redundancy removal by using a sequence clustering software to obtain non-redundant transcripts, and the gene expressive quantity that based on the reads count were done at the same time by the software Corset [23].. These non-redundant transcripts were named unigenes. Bowtie was used to map the clean reads back to the assembled transcripts [24]. Transcripts with ≤70% mapping coverage were discarded from the final assembly.

### Gene function annotations and classifications

Swiss-Prot, Cluster of Orthologous Groups (COG), and Kyoto Encyclopedia of Genes and Genomes (KEGG) databases were used to annotated all the unigenes with local BLAST programs (E value < 1.0E^-5^). For the Swiss-Prot annotation results, the Blast2GO program was applied to classify the unigenes based on the Gene Ontology (GO) terms [25], and the WEGO tool was used to draw the GO tree [26]. The COG database was also used to predict the functional classification of the unigenes. All unigenes were matched with the KEGG pathway database by using the BLASTx to predict the pathway in which the unigenes participated (E value < 1.0E^-5^).

### Identification of differentially expressed genes (DEGs)

Differentially expressed genes (DEGs) of different libraries were analyzed using the fragments per kb of exon region in a given gene per million mapped fragments (FPKM) method and were identified with edgeR package [27]. FPKM was used to determine the number of mapping reads for every unigene and to assess the unigene expression levels. Negative binomial distribution methods were applied to calculate the p values. Benjamini-Hochberg methods were chosen to adjust for the multiple tests. The unigenes were significantly and differentially expressed when they had a p-value < 0.0001 and a false discovery rate (FDR) < 0.05. GO functional enrichment and KEGG pathway enrichment analysis of DEGs were conducted via comparison with the whole-transcriptome background using the formula described in previous studies (Bonferroni-corrected p-value ≤ 0.05) [28]. Note that the database that used for KEGG and GO mapping is derived from kobas database (http://kobas.cbi.pku.edu.cn/), and the mapping of the differentially expressed gene is performed by kobas software [29].

### Validation by real-time quantitative RT-PCR

Thirteen unigenes were chosen to confirm their involvement in the response to high temperature using RT-qPCR method. Primer Premier 6.0 software was used to design gene-specific primers based on the selected unigene sequences (see S1 Table). RT-qPCR was conducted on an ABI Stepone plus System using Fast Start Universal SYBR Green Master Mix (Roche). The PCR amplifications were as follows: 95°C for 3 min, followed by 40 cycles of 95°C for 15 s, and then 60°C for 30 s. A melting curve was obtained from 60°C to 95°C by increasing the temperature stepwise by 0.5°C every 5 s. This step was performed to test the specificity of the amplified product. The expression levels of selected DEGs were normalized by comparing with two internal reference genes, HIS3 and GADPH [30]. Their relative expression levels were calculated via the 2−ΔΔCt method. All RT-qPCR experiments were repeated in three biological replications and in three technical replications.

## Results

### *R*. *obtusum* “Yanzhimi” shows better performance of high leaf survival rate and photosynthesis protection under heat stress

**Fig 1:**
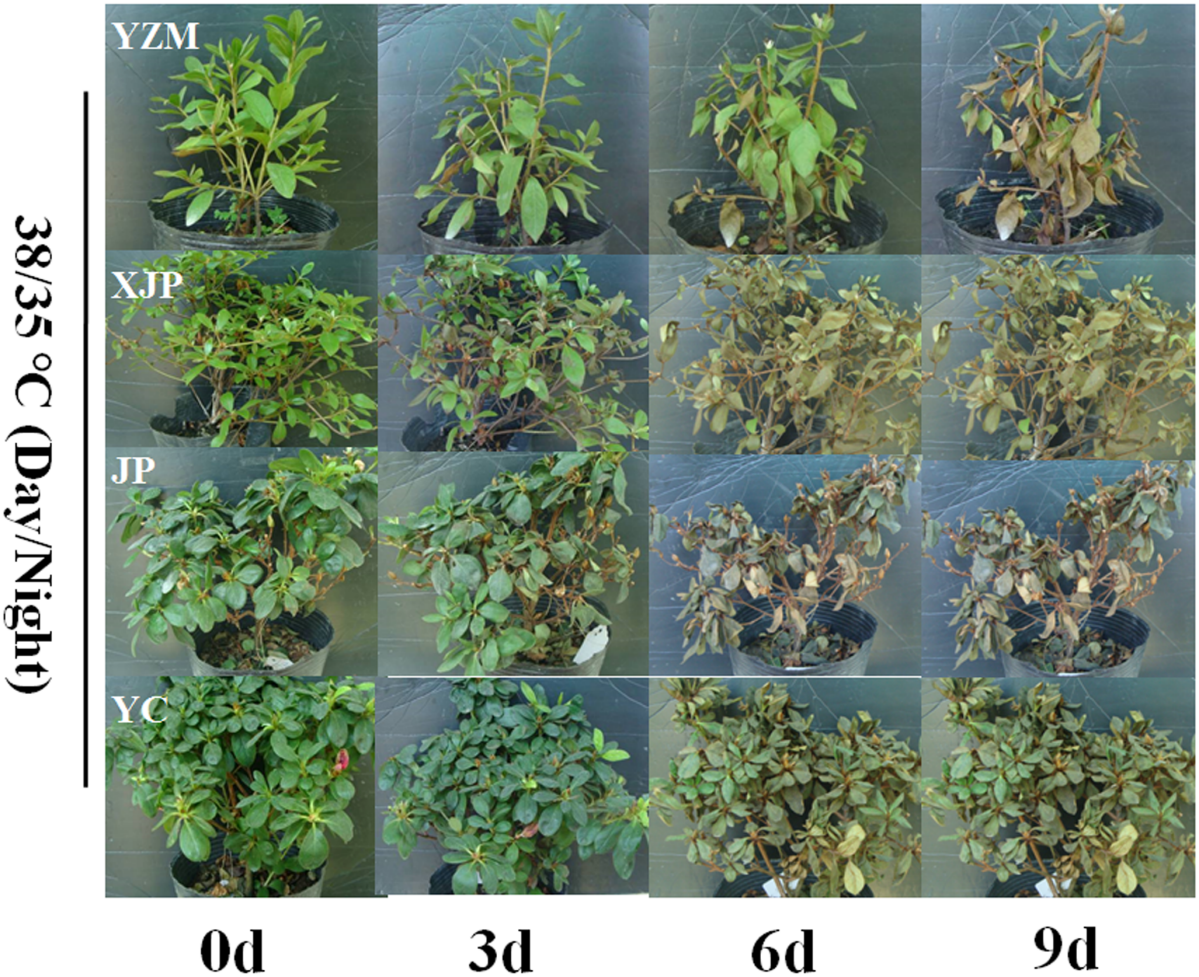
Phenotype of four rhododendron cultivars under heat treatment. 38°C/35°C (day/night) treatment of four rhododendron cultivars. YZM, *R*. *obtusum* “Yanzhimi”; XJP, *R*.*hybridum* “Xiajinpao”; JP, *R*.*hybridum* “Jinpao”; YC, *R*.*hybridum* “Yanchun”.

After 3 days of treatment under 38°C/35°C, all the leaves of “Xiajinpao”, “Jinpao”, and “Yanchun” were withered yellow, and the plants died. In all gradients and high temperature treatments, “Yanzhimi” became pale and showed wilting symptoms after exposure to 38°C/35°C for 6 and 9 days, but grew well under other high temperature stress conditions (Fig 1).

The chlorophyll fluorescence kinetic parameters in the four species of rhododendrons were measured using the imaging-PAM system to estimate the effect of heat stress on photosynthesis and explore the relationship between heat tolerance and photosynthetic activities. Three parameters, including the maximum photochemical efficiency of photosystem II (*F*v/*F*m), efficiency of excitation capture of open PSII center (*F*v’/*F*m’), and initial fluorescence (*F*o), were selected for further investigation.

**Fig 2:**
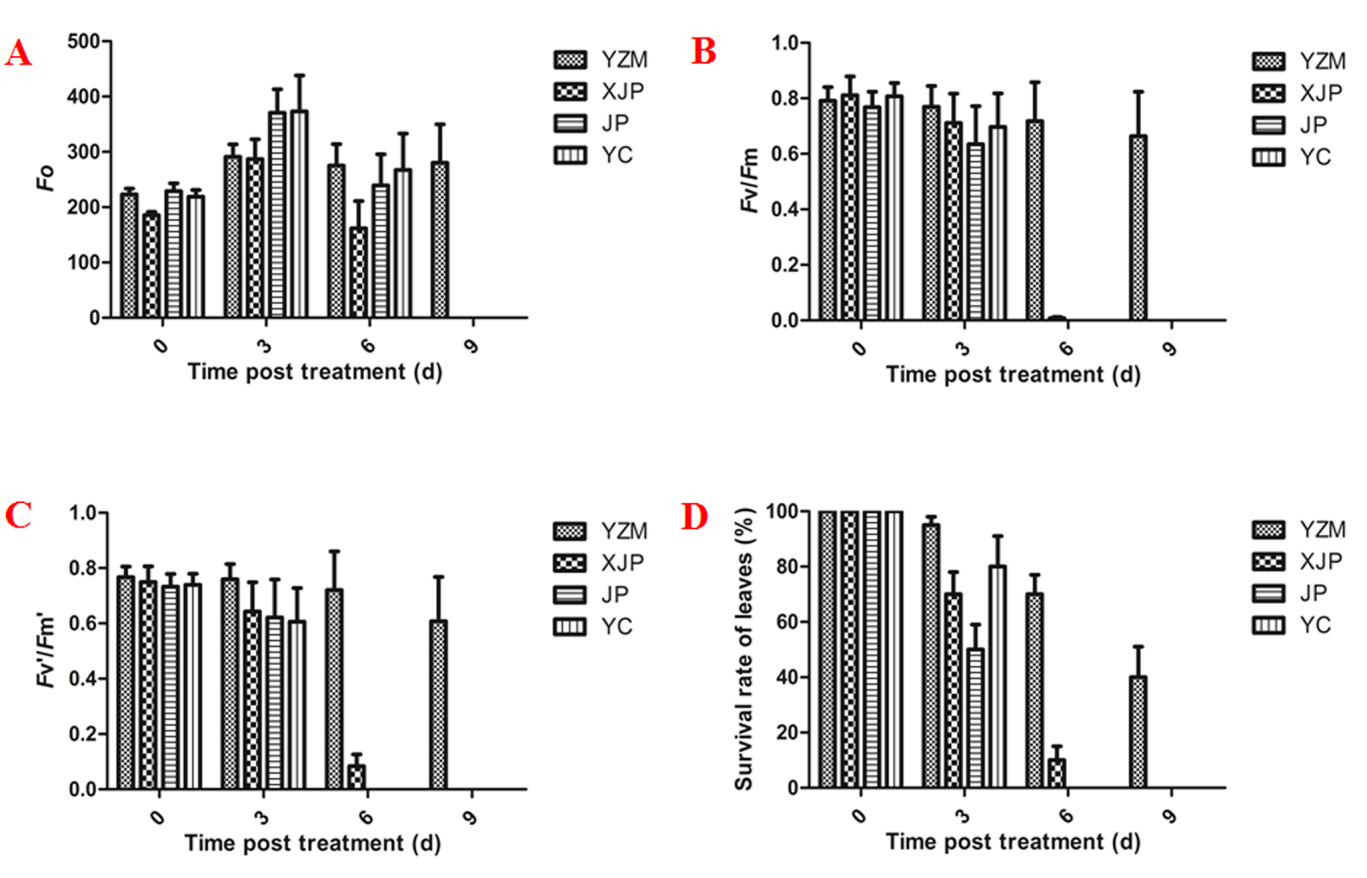
Survival rates of leaves and status of the ChlF parameters. A. *F*o; B. *F*v/*F*m; C. *F*v’/*F*m’; D. Survival rates of leaves. YZM, *R*. *obtusum “Yanzhimi”*; XJP, *R*.*hybridum* “Xiajinpao”; JP, *R*.*hybridum* “Jinpao”; YC, *R*.*hybridum* “Yanchun”.

Under 38°C/35°C treatment, the phenotype of rhododendron was severely damaged and showed diverse differences. Fig 2a shows that the *F*o value increased at the 3-day time point and decreased after 6 and 9 days in the four cultivars. The 38°C/35°C temperature treatment more severely affected the “Jinpao” and “Yanchun” plants as compared with “Yanzhimi” after 3 days. The *F*o value decreased rapidly to an undetectable amounts at the 6-day time point, thereby indicating that the leaf photosynthetic apparatus was irreversible damaged (Fig 2a).

*Fv*/*Fm* showed a decreasing trend in the four rhododendrons. Except for “Yanzhimi”, the values drastically decreased after 3 days and were undetectable after 6 days. The *Fv*/*Fm* of “Yanzhimi” also decreased from 0.79 to 0.77, 0.718, and 0.66 after 3, 6, and 9 days, respectively (Fig 2b). *F*v'/*F*m' exhibited a similar profile to *F*v/*F*m in the four varieties, as shown in Fig 2c.

Increased *F*o coupled with decreased *F*v/*F*m and *F*v'/*F*m' reflects the structural damage of the thylakoid membrane and the inactivation of PSII reaction center at high temperature. Changes in the chlorophyll fluorescence index are negatively related to plant tolerance under high temperature stress. The survival rate of leaves shown in Fig 2d corresponded to the status of the ChlF parameters. “Yanzhimi” showed a slower decrease in ChlF parameters and higher survival rate of leaves than the other three rhododendrons, which indicated than “Yanzhimi” had the strongest protective system that maintains PSII and the best heat tolerance.

### Illumina sequencing and *de novo* assembly

**Fig 3:**
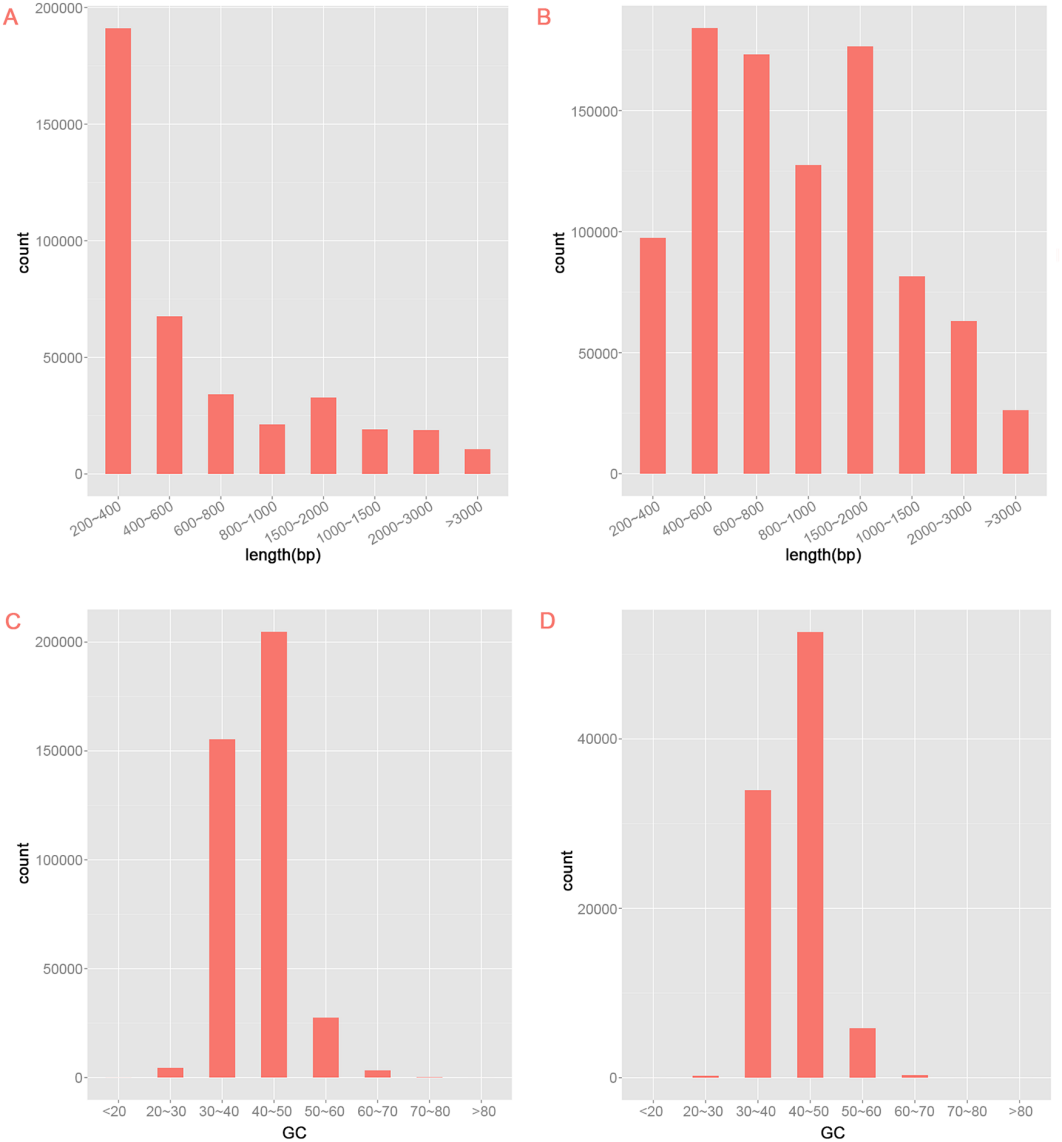
Length distribution and GC content of the assembled transcripts and unigenes.

Nine cDNA libraries were generated with mRNA from the three sample groups: control group (grown at 25°C), group1 (heat treatment exposed at 40°C for 4 h), and group2 (heat treatment exposed at 40°C for 24 h) for the full expanded leaf. These cDNA libraries were then subjected to Illumina deep-sequencing. Clean reads with 4,349,881,212, 4,122,064,627, and 4,508,391,358 nucleotides were obtained from the control, group1, and group2, respectively (Table 1). The clean reads were >114 Gb (total length), which was equivalent to ~160 fold coverage of the genome of *R*. *obtusum* (about 0.7 Gb). All clean reads were *de novo* assembled with the Trinity method because *R*. *obtusum* does not have an appropriate reference genome sequence. Most of the reads could be mapped back to the assembled transcripts. A total of 325,429,240 clean reads were assembled into 395,561 transcripts and then clustered into 92,463 unigenes with a total unigene length of 97,052,187 bp. The length of the unigenes ranged from 201 bp to 15,666 bp with a unigene N50 size of 1,265 bp (Table 2). The most abundant unigene distribution ranged from 400–600 bp to 1,000–1,500 bp, which accounted for 18,126 (19.60%) and 17,661 (19.10%) unigenes, respectively. A total of 57,676 unigenes (57.76%) were within the length range of 401–1000 bp; 32,130 unigenes (34.75%) were in the length range of 1,001–3,000 bp. A total of 2,657 unigenes (2.87%) had lengths of >3,000 bp. The average length of unigenes was larger than those in the transcripts (Fig 3a and 3b). For the GC content, most transcripts and unigenes were distributed within the range of 40%–50% (Fig 3c and 3d), which is identical to the GC content of each sample’s clean data in Table 1.

**Table 1:** Summary of *R*. *obtusum* ‘Yanzhimi’assembled transcripts and unigenes properties.

Sample	raw_reads	clean_reads	raw_bases	clean_bases	Q30(%)	%GC
0h_4	25,837,332	22,846,888	9.37E+09	7.98E+09	97.00401	46.5
0h_6	55,402,556	49,908,900	2.01E+10	1.75E+10	97.30411	47
0h_7	25,511,120	22,563,320	9.25E+09	7.9E+09	97.12327	46.5
4h_1	42,939,396	38,753,828	1.56E+10	1.36E+10	97.25786	47
4h_3	25,631,036	23,100,858	9.3E+09	8.1E+09	97.42258	46.5
4h_6	44,840,326	40,690,620	1.63E+10	1.43E+10	97.4354	48
24h_3	38,653,096	35,377,014	1.4E+10	1.24E+10	97.61504	45.5
24h_4	54,343,006	49,226,880	1.97E+10	1.73E+10	97.4226	46.5
24h_6	48,396,388	42,960,932	1.76E+10	1.5E+10	96.92817	46.5

**Table 2:** The size distribution of transcripts and unigenes of rhododendron transcriptome. Size distribution of Illumina sequencing transcripts(left). (B) Size distribution of unigenes (right).

Type	Transcript	Unigene
N50	1,190bp	1,265 bp
N90	297 bp	551 bp
Average length	602 bp	602 bp
Max length	15,666 bp	15,666 bp
Min length	201 bp	201 bp
Total bases	291,988,981 bp	97,052,187 bp
Total contigs	39,5561	92,971
GC_content	30.06%	30.06%
GC_content_max	81.74%	68.80%
GCcontent_min	8.02%	17.54%

### Functional annotation of the assembled transcriptome

To predict and analyze the function of the assembled unigenes, we assessed the non-redundant sequences using a BLASTX search against the following databases: Swiss-Prot (a manually annotated and reviewed protein sequence database), protein family database (Pfam), Gene Ontology (GO), cluster of orthologous groups (COG), Kyoto Encyclopedia of Genes and Genomes (KEGG) and Orthologous Groups of proteins (EGGnog). After the analysis, 38,724 (41.88%), 24,603 (26.61%), 34,003 (36.77%), 31,769 (34.35%), 27,514 (29.75%), and 27,956 (30.23%) unigenes returned BLAST results and showed identity with the sequences in SwissPro, Pfam, GO, COG, KEGG, and EGGnog databases, respectively (Table 3). Overall, 40,518 (43.82%) unigenes were significantly matched to the known genes in the above-mentioned public databases (S2 Table).

**Table 3:** Functional annotation of rhododendron unigenes by sequence similarity search to Swiss-Prot, Pfam, GO, COG, KEGG and EGGnog database.

Database for annnotation	Number of unigene annotated	Ratio(%)
Swiss-Prot	38,724	41.88%
Pfam	24,603	26.61%
GO	3,400	36.77%
COG	31,769	34.35%
KEGG	27,514	29.75%
EGGnog	27,956	30.23%
Total Unigenes	92,462	100%

**Fig 4:**
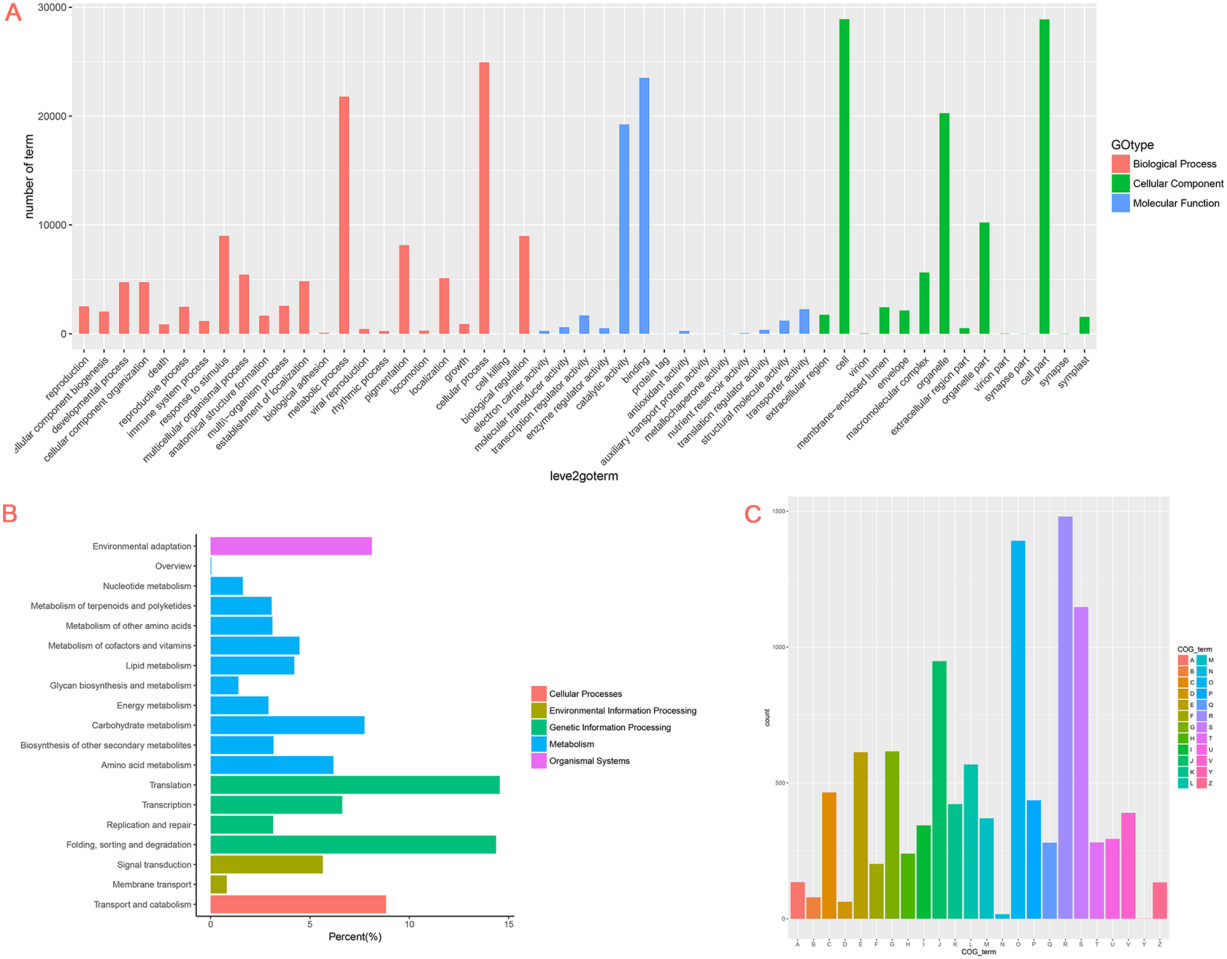
GO, COG, and KEGG pathway classifications of the *R*. *obtusum* transcriptome. A. Classifications are shown in 3 principal categories and 51 functional groups. B. Distribution of each KEGG pathway number against the KEGG database. Each color represents a KEGG pathway. C. COG functional classification of the *R*. *obtusum* transcriptome. The 24 COG category names are indicated, A, RNA processing and modification; K, Transcription; L, Replication, recombination and repair; B, Chromatin structure and dynamics; D, Cell cycle control, cell division, chromosome partitioning; Y, Nuclear structure; V, Defense mechanisms; T, Signal transduction mechanisms; M, Cell wall/membrane/envelope biogenesis; N, Cell motility; Z, Cytoskeleton; W, Extracellular structures; U, Intracellular trafficking, secretion, and vesicular transport; O, Posttranslational modification, protein turnover, chaperones; X, Mobilome: prophages, transposons; C, Energy production and conversion; G, Carbohydrate transport and metabolism; E, Amino acid transport and metabolism; F, Nucleotide transport and metabolism; H, Coenzyme transport and metabolism; I, Lipid transport and metabolism; P, Inorganic ion transport and metabolism; Q, Secondary metabolites biosynthesis, transport and catabolism; R, General function prediction only; S, Function unknown.

The use of GO terms for functional categorization was performed according to the Swiss-Prot annotation. The main GO terms included biological process (BP), cellular component (CC), and molecular function (MF). Based on the sequence homology, 92,463 unigenes were mainly categorized into 51 functional groups (Fig 4a). The GO analysis indicated that many identified genes were associated with various biological processes and molecular functions under heat stress. In the category of BP, the largest groups were associated with cellular and metabolic processes. About 23,106 unigenes were annotated in the metabolic process category, which allowed the identification of novel genes involved in the secondary metabolism pathways during heat acclimation. For the MF category, the unigenes with binding and catalytic activities comprised the largest groups. For CC, the top three largest categories were cell, cell part, and organelles (Fig 4a).

To obtain a better understanding of the biological functions of the unigenes, we used the annotated sequences to search in the KEGG database. Among the 92,463 unigenes, 27,514 had significant matches and were assigned to 121 KEGG pathways (Fig 4b, S3 Table). The most strongly represented pathways for these genes were the metabolism pathways; 767 unigenes out of total 1910 metabolism-related unigenes were enriched under 24 h of heat treatment (Fig 4b, S3 Table). These annotations served as a basis for investigating the processes and pathways involved in the response to heat stress.

A total of 23,514 unigenes were subdivided into 24 COG classifications to categorize the orthologous gene products (Fig 4c). Among these classifications, the cluster of “general function prediction only” (R group) represented the largest group, followed by the “posttranslational modification, protein turnover, chaperones” (O group). The two categories involving “cell motility” (N group) and “nuclear structure” (Y group) represented the smallest COG classifications.

### DEGs among the heat stress treatment and control in rhododendron

Plants require an effective and quickly response to adapt to the heat stress and survive in high temperatures. This rapid reaction might predominantly rely on a gene network involving TFs, protein kinases, plant hormone signal transduction pathways, and ROS signaling. For the executive DEG analysis, all Illumina sequencing biological repeat samples were compared, and sample 0h-6 showed a relatively low correlation with the 0h-4 and 0h-7 samples. Hence, 0h-6 achieved an accurate DEG result (S1 Fig). Subsequent analysis showed that many rhododendron unigenes were significantly affected by the high temperature treatment. We identified 8,395 unigenes as DEGs, including 5,828 up-regulated unigenes and 2,557 down-regulated unigenes at 4 h; whereas 10994 unigenes were classified as DEGs, including 6,140 up-regulated unigenes and 4,854 down-regulated unigenes at 24 h. Control and heat stress samples were identified using a heat map (S2 Fig).

HSPs are environmentally induced proteins that play a role in acquired stress tolerance and enable the plants to cope with heat and other environmental stresses. HSPs also operate and collaborate with other stress response mechanisms and functional components to reduce cellular damage. Analysis of the transcriptome profile in plants after heat treatment indicated that the HSP family plays a central role in the response to heat stress. HSPs families, including HSP100, HSP90, HSP70, HSP60, and sHSPs, are involved in folding and assembling of the protein, maintaining protein stabilization, activating the protein, and degrading the protein in many normal cellular processes and under stress conditions. In this study, 109 DEGs of the HSPs family were detected in the rhododendron transcriptome. These genes showed significant changes under heat treatment (S2 Table). The genes of high and middle molecular weight HSPs, including HSP70, HSP90, and HSP83, were similar to other *de novo* assembled transcriptomes under heat stress [31]. HSP90 (C18696) showed great differential expression under 4-h and 24-h heat treatments, which represent log2 ratios of 13.32 and 9.72, respectively.

### Identification of TFs and target genes in *R*. *obtusum* “Yanzhimi”

TFs are important in gene transcriptional expression regulation network by affecting the downstream expression of many genes. TFs can guide many different biological processes, including secondary metabolism, cell division, and cell differentiation. Therefore, the identification of TFs and target genes will provide a better understanding of the regulatory networks of genes in the heat tolerance of *R*. *obtusum*.

**Fig 5:**
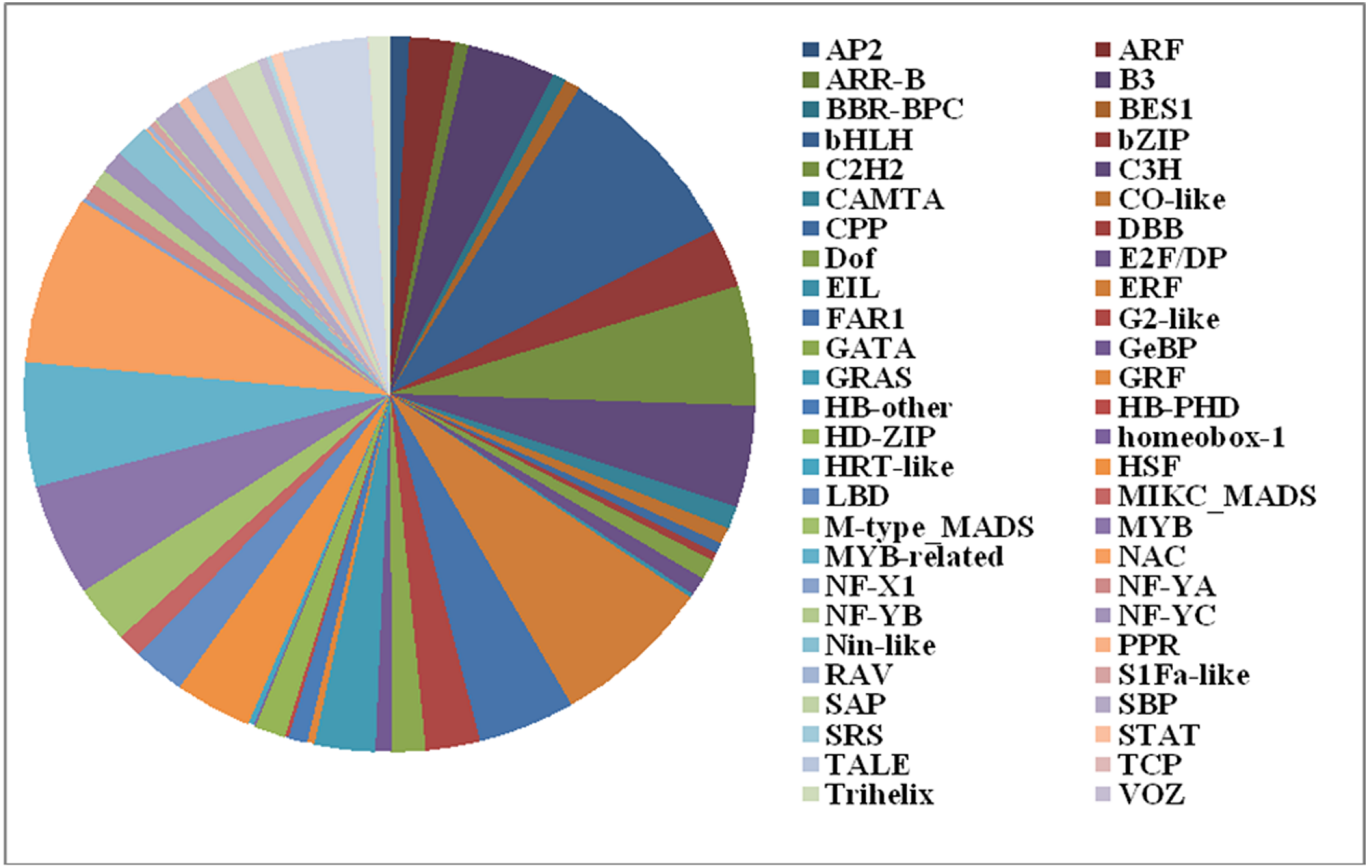
Transcript gene family classification of *R*. *obtusum* transcriptome.

A total of 973 protein sequences were obtained after ORF prediction and default filter screening. These sequences can be divided into 57 TF families, including the widely reported gene family involved in abiotic stress tolerance; for instance, several DREB/CBFs, MYBs, NACs, and HSFs were characterized in our transcriptome (Fig 5 and Table 4). Among these families, the 33 annotated HSFs can be subdivided into 3 subgroups including 22 HSFAs, 10HSFBs, and 1 HSFC. Their differential expression profiles are shown in S2 Table.

**Table 4:** The number of unigenes identified as transcription factors in rhododendron leaves. 24 h vs ck, Changed log2 ratio of Rhododendron exposed to 40°C for 4 h. B. Changed log2 ratio of Rhododendron exposed to 40°C for 24 h.

Unigene	Annotation	TFfamily	Top Hit	Log2 ratio	
				24h vs ck	4h vs ck
C44421	AP2	AP2/EREB	AtAP2	3.38	4.76
C44420	AP2	AP2/EREB	AtAP2	3.13	6.08
C61022	DRE element binding	AP2/DREB	AtDREB1D	3.61	4.11
C46630	DRE element binding	AP2/ DREB	AtDREB1C	3.99	4.94
C41140	DRE element binding	AP2/ DREB	AtDREB2C	7.32	7.32
C62227	DRE element binding	AP2/ DREB	AtDREB2A	8.23	3.26
C62226	DRE element binding	AP2/ DREB	AtDREB2A	9.11	3.40
C85885	Ethylene-responsive	AP2/ERF	AtERF17	8.47	8.00
C85886	Ethylene-responsive	AP2/ERF	AtERF17	7.41	7.60
C85726	Ethylene-responsive	AP2/ERF	AtERF53	3.61	2.07
C56488	Ethylene-responsive	AP2/ERF	AtERF71	5.23	2.31
C17302	Ethylene-responsive	AP2/ERF	OsERF1	10.62	9.71
C6549	Ethylene-responsive	AP2/ERF	AtERFL1	3.24	0.69
C87291	bZIP	bZIP	AtbZIP44	4.82	3.23
C84155	bZIP	bZIP	AtBZIP61	4.50	-0.11
C69287	bZIP	bZIP	SlHY5	6.54	5.60
C69286	bZIP	bZIP	SlHY5	9.06	6.78
C24020	E2F	E2F	AtE2FE	6.41	2.90
C51101	GATA	GATA	AtGATA8	2.26	-1.85
C52862	Heat stress transcription	HSF	AtHFA6B	5.64	8.90
C75881	Heat stress transcription	HSF	AtHFB2A	2.57	3.51
C80422	Heat stress transcription	HSF	AtHSFA2	9.52	14.19
C63903	Heat stress transcription	HSF	AtHSFA3	6.52	5.41
C63905	Heat stress transcription	HSF	AtHSFA3	8.17	7.26
C63904	Heat stress transcription	HSF	AtHSFA3	6.62	8.25
C63906	Heat stress transcription	HSF	AtHSFA3	7.71	7.75
C60158	MADS-box	MADS	AtFLC	5.90	2.65
C40226	MADS-box	MADS	OsMADS1	6.69	5.54
C31525	MYB domain-containing	MYB	SlMYB1R1	2.91	2.03
C61539	MYB domain-containing	MYB	ZmMYB1	3.79	1.11
C52500	MYB domain-containing	MYB	AtMYB12	4.91	-2.57
C14251	MYB domain-containing	MYB	AtMYB21	7.74	3.63
C30941	MYB domain-containing	MYB	NA	3.31	0.75
C81022	NAC domain-containing	NAC	AtNAC2	5.49	2.61
C45824	NAC domain-containing	NAC	AtNAC2	4.20	4.81
C61024	NAC domain-containing	NAC	AtNAC29	7.00	6.43
C14207	NAC domain-containing	NAC	AtNAC72	2.26	1.47
C65230	NAC domain-containing	NAC	AtNAC8	2.21	1.40
C72455	Ethylene-responsive	NAC	AtABR1	4.66	1.53
C86364	WRKY	WRKY	AtWRKY33	3.30	3.04
C86362	WRKY	WRKY	NA	8.10	7.27
C51799	WRKY	WRKY	AtWRKY51	2.44	2.36
C52443	WRKY	WRKY	AtWRKY42	7.39	5.48
C47837	WRKY	WRKY	AtWRKY44	5.19	2.00
C85009	C3H1domain-containing	Znf	OsC3H17	3.10	1.53
C32262	C3H1domain-containing	Znf	AtC3H2	6.76	6.22
C43242	C3H1domain-containing	Znf	OsC3H13	3.69	4.28
C60868	C3H1domain-containing	Znf	AtC3H48	6.36	6.94
C65939	Zinc finger protein	Znf	AtZAT10	5.59	5.94
C36936	Zinc finger protein	Znf	AtZAT8	6.99	8.89
C36935	Zinc finger protein	Znf	AtZAT11	8.34	8.60
C36937	Zinc finger protein	Znf	AtZAT8	10.99	9.83

The number of TFs in *R*. *obtusum* was lower than in the model plant. In *Arabidopsis*, 1922 known genes encode TFs that can be divided into 64 gene families. In rice, 2025 TF genes were annotated in PlnTFDB [32]. The expression levels of AP2/EREB, AP2/ERF, HSF, MYB WRKY, ZNF, and other gene family members were screened from the rhododendron transcripts by using log2 ratio> 2.0 and P-value <0.001 as the differential expression thresholds. We especially focused on the validation of HSF gene family members at 24-h heat treatment stage. AtDREB1D (C61022), AtDREB1C (C46630), AtDREB2C (C41140), AtDREB2A (C62227), and AtDREB2A (C62226) were reported by the DREB class of TFs. These genes were also reported as important TFs involved in cold, drought, high temperature, and high salt stress in *A*. *thaliana*. Fifty-two TFs were finally selected with up-regulated log2 ratio >2.0 and FPKM >10 filtering. These TFs are possible regulators that positively transcriptionally regulate the heat responsive genes in rhododendrons. The expression level of C62226 gene showed the highest up-regulation, and the log2 ratio was as high 9.11 at 24h. This dramatic response indicated that DREB plays an important role in rhododendron under heat stress. Moreover, HSFB2A (C75881), HSFA2 (C80422), HSFA3 (C63903, C63904, C63905, C63906), and HSFA6b (C52862) also show greatly up-regulated changing fold under 4-h or 24-h heat stress treatments, respectively, thereby showing the involvement of the HSF gene family in heat response. The expression of C80422, which hits AtNAC2 gene in *Arabidopsis* was high, and the log2 ratio was 9.52 at 24 h (Table 2).

### Validation and expression pattern analysis

**Fig 6:**
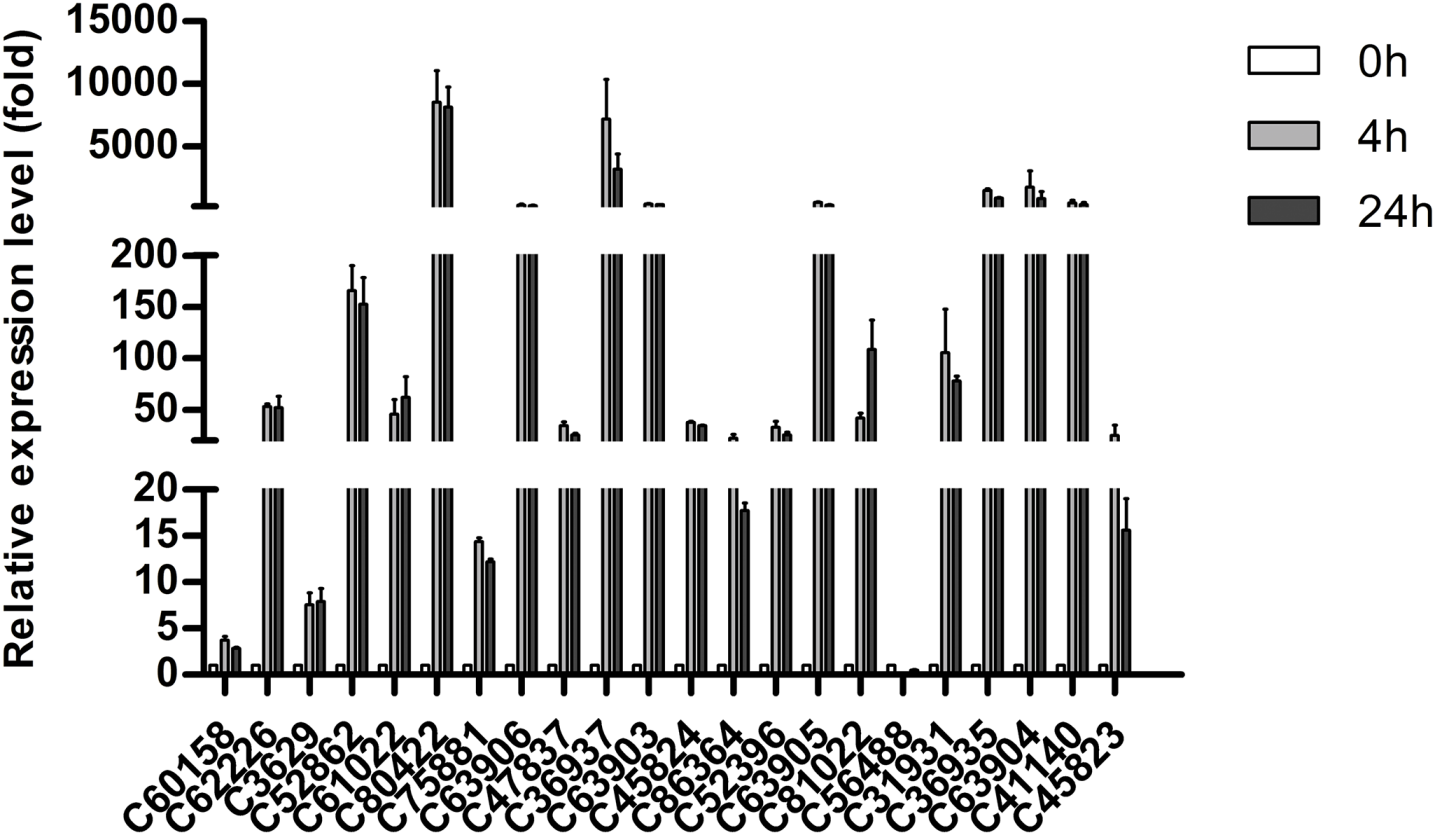
qRT–PCR analysis of TFs in response to different heat stresses. Three bio-replicates and tech-replicates were performed. Data are presented as means±SD.

Twenty-two candidate DEGs were selected, and their expression profiles were compared between control and heat stress samples using quantitative real-time (qRT) PCR to further validate the reliability of the Illumina sequencing read analysis and to detect the expression of some candidate regulators. Among the chosen DEGs, dehydration-responsive element-binding protein 2A (C62226), dehydration-responsive element-binding protein 2C (C41140), dehydration-responsive element-binding protein 1D (C61022), heat stress TFA2 (C80422), heat stress TF A3 (C63903, C63904, C63905, and C63906), heat stress TFA6b (C52862), Zinc finger protein ZAT8/ZAT10/ZAT11 (C31931, C36935, C36936, and C36937), NAC domain-containing protein NAC2/NAC29 (C3629, C81022, C45823, and C45824), and WRKY TFs (C86364) were related to the response to abiotic stresses. The expression patterns of all these DEGs obtained through qRT-PCR data were consistent with the high-throughput RNA-sequencing (RNA-Seq) data, thereby confirming that the Illumina results were reliable (Fig 6). As shown in Fig 6, most of the selected TFs showed a significantly up-regulated expression pattern under 4 h and 24 h heat treatments. The highest expression levels of many DEGs were observed at 4 h as compared with 0 and 24 h, thereby indicating that these TFs responded to high temperature at the early stage of heat stress and subsequently accumulated to exhibit their regulatory effect.

### Effect of heat stress on photosynthesis-related unigenes

**Fig 7:**
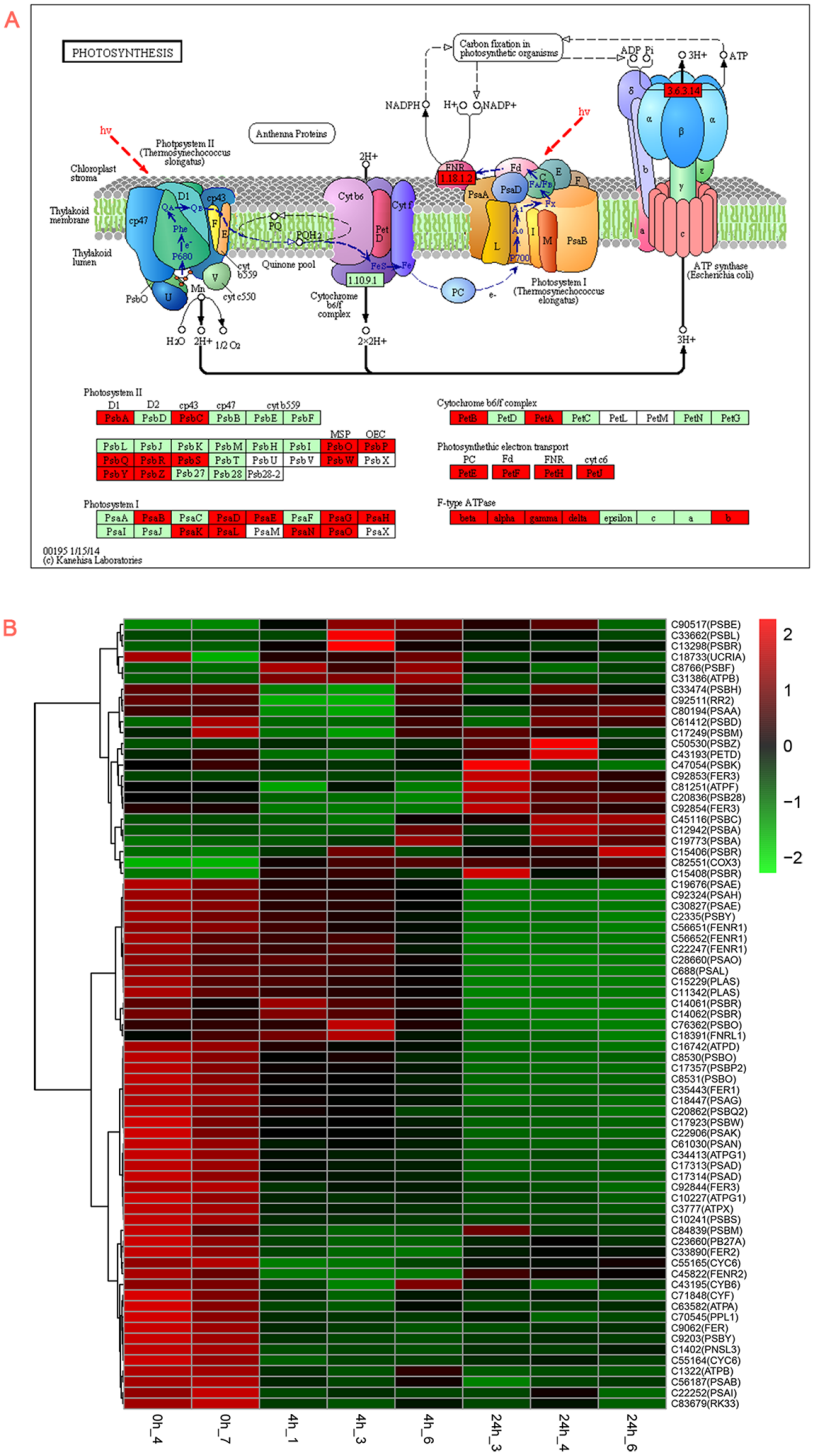
Enrichment of photosynthetic passway and relative unigene cluster heatmap. A. KEGG-illustrated photosynthetic passway was significantly enriched in 24-h heat treatment compared with the control. Red color indicates that the unigenes were up-regulated significantly, green color indicates that the unigenes were down-regulated significantly, and gray color indicates that the unigenes were unchanged. B. Heat map cluster of photosynthesis passway unigenes in rhododendron.

Based on the KEGG pathway enrichment analysis, we found that the photosynthesis pathway was significantly changed under 4-h or 24-h heat treatment. In this pathway, 48 unigenes showed differential expressions out of 77 characterized unigenes in rhododendron transcriptome (Additional file 4). These DEGs covered all the sections of this pathway, including PSI, PSII, cytochrome b6/f complex, photosynthetic electron transport, and F-type ATPase. The activity of PSII is restricted by a variety of environmental stresses, and the ROS produced in this process activates the *psbA* mRNA [33]. Mattoo et al. [34] reported that both electron transport and the synthesis of ATP are important for the synthesis of D1 in *Spirodela oligorrhiza*. Studies on intact chloroplasts from spinach showed that the level of stromal ATP is correlated with the light-dependent synthesis of the D1 protein in intact chloroplasts [35]. D1 (psbA) and 5 ATPases (beta, alpha, gamma, delta, and b) were up-regulated in this process, thereby supporting the results of the previous study (Fig 7). Photosynthesis under heat stress might be hindered by the reduction of soluble proteins, Rubisco binding proteins, large-subunits (LS), small-subunits (SS) of Rubisco in darkness, and the increase in amounts of these proteins under light [36].

## Discussion

Considering that *Rhododendron* consists of non-model and woody plants, current data on the molecular and genetic properties of species in this genus are severely insufficient. Only a few transcriptome databases were established for *R*. *latoucheae* Franch. and *R*. *fortunei* [37, 38]. These databases focused on root nutrition adoption and development of new EST-SSR markers, respectively, but not on the abiotic stress. The lack of reference genomic and transcriptome data restricted the research on rhododendron, especially the molecular mechanism underlying its complex traits. As a feasible and economical technology for creating relatively comprehensive sequence data in a short time, transcriptome RNA sequencing technology has become popular in plant research [39–43]. In the present study, 92,463 unigenes were assembled. The obtained sequence data can serve as a basis for further studies on gene cloning, expression analysis, and SSR marker development.

By comparing the morphological and physiological indexes in different cultivars, we screened a heat tolerant material to identify the related key genes that determine its traits. The up-regulated TFs screened in *R*. *obtusum* “Yanzhimi” transcriptome, including HSFs, DREBs, ZATs, and NACs, belonged to important gene families that participate in abiotic stress tolerance, and their homologs were functionally characterized in the model species. For instance, the constitutive expression of the C2H2-EAR zinc finger protein Zat10 in *Arabidopsis* elevated the expressions of reactive oxygen-defense transcripts [44], and the homologous gene named C65939 in rhododendron transcriptome was up-regulated. In our transcriptome, four HSFA3 homologous genes were identified and named as C63903, C63904, C63905, and C63906, and their expression levels were increased by 10,000-fold after heat treatment for 24 h. Considering that only one gene copy of HSFA3 was found in *A*. *thaliana* and rice, the four homologous unigenes detected in our transcriptome are greater in expression than in the model plants. This finding can be explained by several reasons. First, the horticultural variety *R*. *obtusum* “Yanzhimi” might be highly heterozygous in this locus, resulting in a larger allelic variation. Second, a draft reference genome of the rhododendron plants has not yet been published. Hence, the evolution of ancestral whole genome polyploidization has not yet been confirmed in rhodondendron but has already been established in other basal eudicots, such as *Vitis vinifera* and *Populus* [45, 46]. Some HSFA3 paralogs were found. Third, the heat TF could form the truncated S-HsfA2, which is terminated prematurely by variable shear and binds to the promoter sequence [47]. Whether the rhododendron HSFA3 will pass through this mechanism, which further results in detecting multiple transcripts, still needs to be verified by subsequent experiments.

The homologous unigene AtHSFA2 (C80422) shows a significantly up-regulated expression under 24-h heat treatment (change fold > 16,000). Hence, we presumed that this gene functions positively in the heat tolerance of rhododendron. In *Arabidopsis*, AtHSFA2 directly regulates the chloroplast-localized HSP21 and other heat shock transcript proteins and repairs the thylakoid membrane damage to protect the chloroplasts. This phenomenon is consistent with our findings on its protective activity in the PSII reaction center. As another important member in HSFA subclade gene family, *AtHsfA3* can bind directly with DRE cis-element by AtDREB2A/AtDREB2C, thereby playing an important role in plant cold and drought stress signaling by positively regulating the heat tolerance in *Arabidopsis*. This finding indicated that DREB-HSF-HSP pathway is conserved in plants, and *HsfA3* serves as a converging node in the cross-stress transcriptional expression network [48].

In summary, the relatively low values of Fv/Fm and Fv’/Fm’ under heat stress reveal that the electron transfer at acceptor side of PSII was blocked. Thus, the heat-induced damage of photosynthetic apparatus was relatively lower in *R*. *obtusum* “Yanzhimi” leaves (Fig 2). A series of heat responsive unigenes was characterized in our transcriptome. These unigenes include some important TFs that show significant fold changes under heat stress and are involved in the transcriptional regulation of photosynthetic structure and protective genes. These results are consistent with results of previous studies on grapevine and spinach [10, 31]. Thus, this study provides the candidate unigenes and pathways involved in the heat tolerance, thereby deepening our understanding of the molecular and physiological processes. This study also provided a large amount of basic genomic data for further research.

## Supporting information

S1 Fig Three biological repeats of 3 groups of samples expression level correlation scatter plot. (TIF)

S2 Fig Expression gene hierarchical clustering analysis. The abscissa is the sample, the ordinate is the transcript, and the different colors indicate the different gene expression levels (shown by the logarithmic form of FPKM), and the color is expressed by the blue to red expression. Blue represents a low expression gene, and red indicates a high expression gene.
(TIF)

S1 Table The qRT–PCR primer sequences of selected heat responsive TFs. (XLSX)

S2 Table Summary of all DEGs with their expression profiles and annotation in different databases. (XLSX)

S3 Table KEGG classification of rhododendron unigenes and significantly enrichment pathways of 24 h heat treatment compared with control. (XLS)
