# Supplementary figures and images for: De novo RNA sequencing transcriptome of Rhododendron obtusum identified the early heat response genes involved in the transcriptional regulation of photosynthesis
Source: PLoS One. 2017 Oct 23;12(10):e0186376. doi: 10.1371/journal.pone.0186376 (PMC5653301; doi:10.1371/journal.pone.0186376)

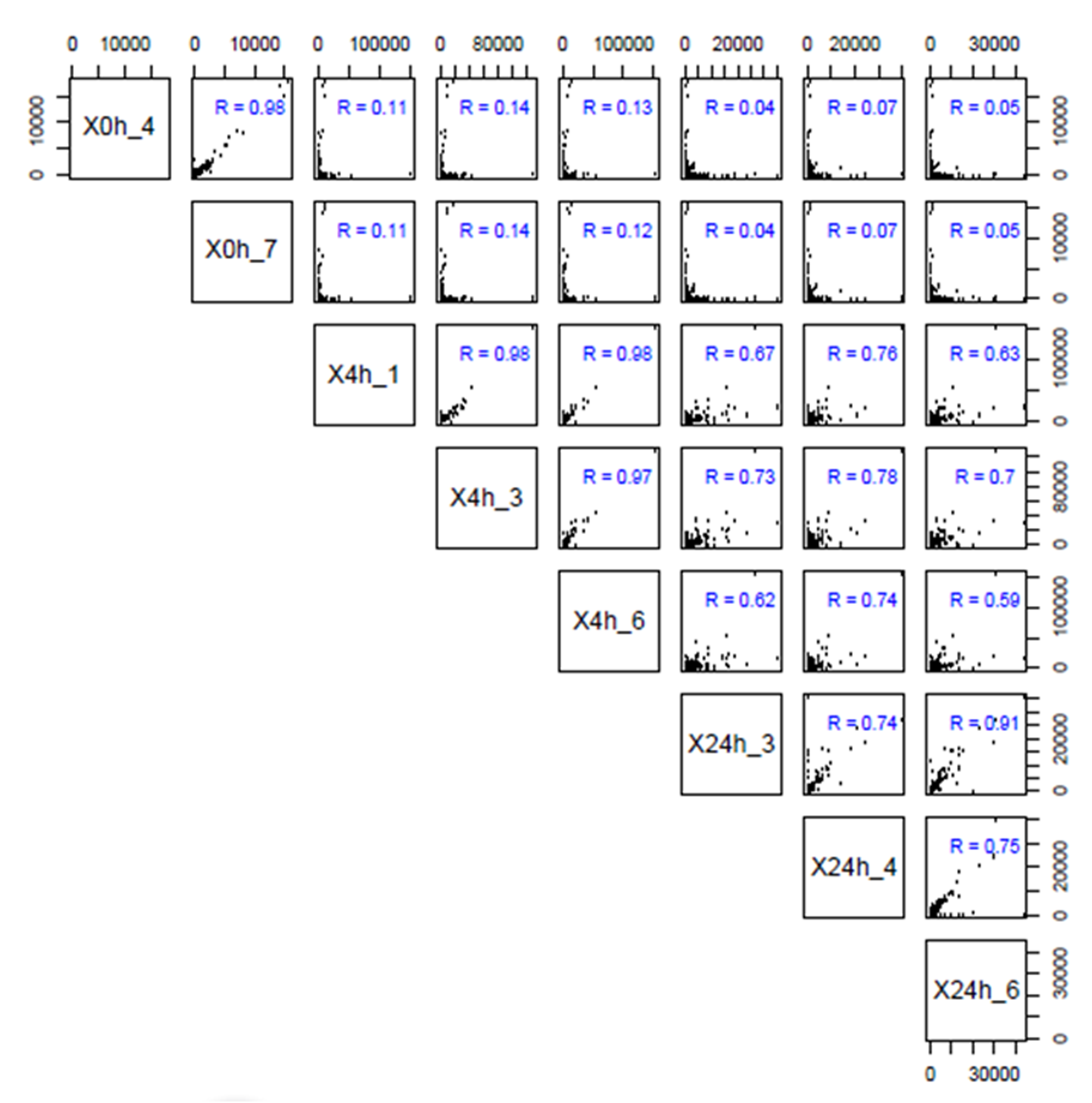

Supplement: S1 Fig — (TIF) [file pone.0186376.s001.tif]

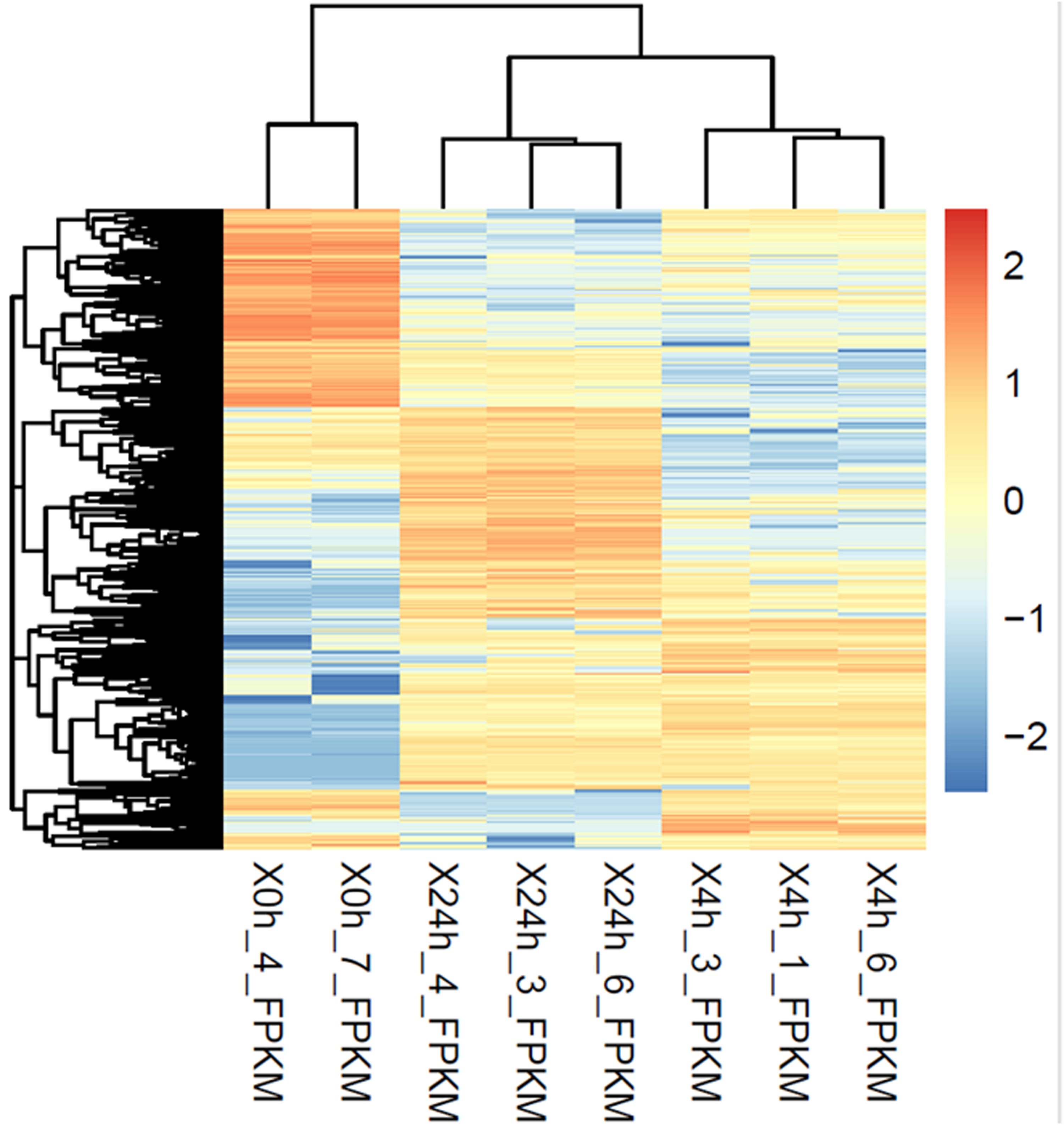

Supplement: S2 Fig — The abscissa is the sample, the ordinate is the transcript, and the different colors indicate the different gene expression levels (shown by the logarithmic form of FPKM), and the color is expressed by the blue to red expression. Blue represents a low expression gene, and red indicates a high expression gene. (TIF) [file pone.0186376.s002.tif]
